# The Effect of Pubertal Stage on the Concentrations of the Novel Adipomyokine, Irisin, in Male Adolescents

**DOI:** 10.4274/jcrpe.galenos.2019.2019.0119

**Published:** 2020-06-03

**Authors:** Demet Taş, Alkım Akman Öden, Sinem Akgül, Ziya E. Metin, Aslı Pınar, Nuray Kanbur

**Affiliations:** 1 University of Health Sciences Turkey Child Health and Diseases Hematology Oncology Training and Research Hospital, Clinic of Pediatrics, Ankara, Turkey; 2 Hacettepe İhsan Doğramacı Children’s Hospital, Clinic of Pediatrics, Division of Adolescent Medicine, Ankara, Turkey; 3 University of Health Sciences Turkey Child Health and Diseases Hematology Oncology Training and Research Hospital, Ankara, Turkey; 4 Hacettepe University Faculty of Medicine, Department of Medical Biochemistry, Ankara, Turkey

**Keywords:** Irisin, male adolescent, puberal stage, body fat percentage, muscle mass

## Abstract

**Objective:**

Irisin is a recently discovered protein and is defined as an adipomyokine. The relation of irisin with carbohydrate metabolism and other hormone parameters have been investigated. However, studies evaluating the relationship between irisin and puberty are limited and inconclusive. The aim was to evaluate serum concentrations of irisin during different pubertal stages in male adolescents.

**Methods:**

The study included normal weight pubertal male adolescents between the ages of 13^6/12^-14^11/12^ who had entered puberty. Fasting serum irisin concentrations were evaluated, and bioelectrical impedance analysis was used to measure body fat ratio (BFR) and fat-free mass (FFM). BFR was also calculated by caliper measurement of subcutaneous fat at the triceps.

**Results:**

Sixty-eight adolescents were enrolled. The number of adolescents in pubertal stage 2, 3, 4 and 5 were n=17 (25%), n=13 (19.1%), n=21 (30.1%) and n=17 (25%), respectively. The median values of the irisin are 8.80, 8.20, 9.15 and 7.24 ng/mL according to the 2-5 pubertal stages, respectively. The levels of circulating irisin did not differ according to the pubertal stage. Additionally, there was no significant relationship between irisin levels and body fat percentage or FFM.

**Conclusion:**

Irisin levels do not differ after the onset of puberty or with progressing pubertal maturation. This study strengthens the evidence that there is no change in irisin concentration as puberty progresses. This may have important implications when using this adipomyokine in the future for diagnosis or treatment of obesity-related diseases.

**What is already known on this topic?**

Irisin is a recently discovered peptide and is defined as an adipomyokine. The amount of fat and muscle tissue and gender affect the release of irisin. The relationship between irisin levels and pubertal stages (2-5) after the onset of puberty has not been studied.

**What this study adds?**

This study adds to the limited evidence available concerning the relationship between pubertal stage and irisin concentrations and strengthens the view that irisin concentrations do not change in response to pubertal progression.

## Introduction

Irisin is a myokine recently described by Boström et al (1) and is derived from the extracellular N-terminal domain of fibronectin type 3 domain-containing-5 (FNDC5), a myocyte transmembrane protein. The transfer of irisin from muscle to circulation after exercise is regulated by peroxisome proliferator-activated receptor-γ co-activator 1α. Irisin also contributes to the regulation of energy consumption and glucose metabolism by influencing the transformation of white fat tissue to brown fat tissue (2).

Some studies (3,4,5) have suggested that irisin is released from muscles in connection with exercise, and muscle mass is the determining factor for the levels of irisin in circulation. However, Pekkala et al (6) reported that irisin levels were not associated with either acute or chronic high intensity or low intensity exercise, and no association was reported between overweight or impaired glucose tolerance and irisin concentrations in the same study. Since high levels of irisin were found in people with obesity, it was also suggested that irisin was released from adipose tissue (7,8). This has led to irisin being considered as an adipomyokine, a hormone released from both muscle and adipose tissues and affects distant organs (9,10).

Several studies have investigated the relationship between irisin and body mass index (BMI), exercise, thyroid function tests, bone metabolism, regulation of blood glucose, and metabolic syndrome in adults (11,12,13,14). Similarly, the relation between irisin and BMI, exercise, weight loss, and metabolic and anthropometric measurements have also been investigated in pediatric age groups (15,16). All of these studies imply that irisin levels depend on the ratio of body fat and muscle mass. Muscle and body fat mass increases with growth during childhood and varies with gender during puberty. When the increase in an individual’s body fat and muscle mass exceeds a critical, personal limit, the hypothalamic-pituitary-gonadal axis is stimulated, and puberty begins (17,18). During puberty, the amount and distribution of muscle and fat mass varies according to gender and pubertal stage. While the fat-free mass (FFM), most of which consists of muscle mass, and body fat are not different between girls and boys in the prepubertal period, at the end of puberty boys have 1.5 times more muscle mass than girls whereas girls have more body fat than boys (19). Since the level of irisin is reported to be associated with both fat and muscle mass (20), irisin levels may also vary along with the pubertal stages in adolescents.

To date, very few studies in children and adolescents have addressed the effects of puberty on irisin levels. In two studies it was concluded that the prepubertal/pubertal stage was not associated with irisin levels. However, in one study, it was reported that the level of irisin was higher in pubertal adolescents than in prepubertal children (21,22,23).

Considering the future utility of irisin in treatment, in relation to many factors such as metabolic or chronic disease and obesity, it may be important to know how concentrations of irisin change according to sex and pubertal stage. The objective of this study was to investigate whether irisin levels differ according to pubertal stages in male adolescents.

## Methods

This cross-sectional analytical study was conducted with eligible participants from the adolescent outpatient clinic. The study was approved by the Research Ethics Committee at Hacettepe University (protocol number: GO 16/721-08, date of approval: 24.11.2016). Written informed consent was obtained from all participants and their parents. Eligible subjects were male adolescents, aged between 13^6/12^-14^11/12^ years, of healthy weight, with no chronic illness, who had entered puberty and were attending well-child care visits. The study focused on male adolescents between 13^6/12^-14^11/12^ years to control for the age variable and to ensure that all participants had entered puberty. Additionally, peak height velocity, minimum body fat ratio (BFR) and maximum FFM are all observed at the age of 14 in male adolescents (24). Those who had a psychiatric, or endocrine disease, were using chronic medication for any reason, were underweight (BMI equal to or under than the 5th percentile), overweight or obese (BMI equal to or higher than the 85^th^ percentile), had exercised a day before the study, were elite athletes, had a special diet, or took food supplements were excluded from the study. In addition, patients with acute infection during the examination, and those with pathological findings related to lipid or glucose metabolism were excluded from the study. Maturation of sexual development was based on Tanner and Whitehouse (25) stages according to pubic hair stage and testis volume. Patients were examined by the same clinician for pubertal staging.

Body weight was measured to the nearest 0.1 kg using a body composition analyzer (Tanita SC-330). Height was measured using a fixed wall-scale to the nearest 1 mm. BMI (kg/m^2^) was used to define healthy weight (5th to 85^th^ percentile), according to age and sex-specific growth reference data (26). FFM (kg) and BFR were measured by the bioelectric impedance analysis (BIA-BFR) technique with Tanita SC-330 (Tanita Corp. Tokyo, Japan). BIA was performed without socks, shoes, and heavy clothing in the morning after eight hours of fasting.

Additionally, BFR was calculated by triceps skinfold thickness measurement. Skinfold thickness was measured with a Harpenden caliper at the tricep, at the middle point between the acromion process and olecranon process on the left arm (27). Subcutaneous adipose tissue was measured by gently pulling the skin and subcutaneous fatty tissue upwards while the patient was standing upright and arms drooping on both sides. The measurement was completed twice and repeated if the difference was more than 1 mm. All skinfold measurements were performed by the same specialist. The body fat percentage was calculated by Triceps Skinfold Thickness (Triceps-BFR) measurement, using the reference values for Turkish children and adolescents (28).

### Irisin Measurement

Adolescents who met the inclusion criteria were invited to the clinic at 08.30-09.00 am after eight hours of fasting to obtain blood samples for serum irisin measurements. Serum was separated and stored at -80 °C until the time of analysis, which was no more than three months in any case. Quantitative measurements of irisin were performed with human FNDC5 enzyme-linked immunosorbent assay (ELISA) kit (Catalog No: E-EL-H2254 Elabscience, Wuhan, China) with a sensitivity 0.10 ng/mL and detection range of 0.16-10 ng/mL and interassay coefficients of variation <6%. Irisin concentrations were expressed as ng/mL.

### Statistical Analysis

Data from the study were analyzed using SPSS 23.0 for Windows, version 23.0 (IBM Inc., Armonk, NY, USA). Descriptive statistics were presented as mean±standard deviations, frequency distributions, and percentages. Chi-square test was used to analyze categorical variables. The normal distribution of variables was tested using visual (histogram and probability graphs) and analytical methods (Kolmogorov-Smirnov or Shapiro-Wilk Test). The variance equation was controlled by the Levene test. One-way analysis of variance was used when parametric test preconditions were met to determine whether there was a significant difference between the three groups and Bonferroni test was used for *post-hoc* tests for double comparisons. The Kruskal-Wallis-H test was used when data distribution was more than two non-parametric data. The relationship between variables was evaluated by Pearson correlation coefficient or the Spearman correlation coefficient as appropriate. Significance was assumed if p<0.05.

## Results

Sixty-eight adolescents were included in this study. The number of adolescents in Tanner stages 2, 3, 4 and 5 were n=17 (25%), n=13 (19.1%), n=21 (30.1%) and n=17 (25%), respectively. Table 1 shows mean body weight, mean height, mean BMI percentile (BMIp), irisin, BFR and FFM values by pubertal stage.

As might be expected a significant positive correlation was found between Triceps-BFR and BIA-BFR (r=0.444 p=0.01) and Triceps-BFR and FFM (kg) (r=0.446; p=0.01).

FFM did not differ between Tanner stages 2 and 3. The increase in mean FFM was significant between Tanner 2/3 and Tanner 4 and increased signidficantly again to Tanner 5. However, the change in mean irisin concentrations by pubertal stage was not statistically significant with no evident trend in concentrations (see Table 2).

Correlation analysis of BMIp, BIA-BFR, FFM, and Triceps-BFR variables for each pubertal stage are given in Table 3. A significant correlation was found between BIA-BFR and Triceps-BFR and BMIp. There was a significant correlation between FFM and Triceps-BFR, BMIp and pubertal stage. Irisin was not found to be correlated with any of the parameters. The inter-correlations between the parameters investigated in this study are shown in Table 4.

## Discussion

To the best of our knowledge, this is the first study to evaluate circulating irisin levels according to pubertal stages (stage 2-5) after the onset of puberty in male adolescents. In the literature, there are only four clinical investigations evaluating circulating irisin levels in which the participants consisted of adolescents, but these studies did not specifically interpret the changes according to pubertal stages. These are briefly reviewed below to build up the background for the discussion of the results of our study.

Al-Daghri et al (29) conducted their research with adolescents between 12 and 15 years of age with healthy body weight and found positive relationships between irisin and fasting blood sugar and high-density lipoprotein cholesterol. Circulating irisin levels of female adolescents were found to be higher than male adolescents and, in a multivariate regression analysis for potential confounders, the irisin levels were independently associated with fasting blood glucose levels predominantly in girls which led the authors to conclude that irisin is a predictor of glucose metabolism which has sexually dimorphic effects in adolescence. The participants were not separated according to pubertal stage and the relationship between irisin and puberty was not mentioned.

Blüher et al (22) evaluated irisin concentrations at baseline and follow-up in obese children and adolescents between 7-18 years of age after a yearlong intervention. At baseline, they did not find any significant relationships between irisin levels and age, gender, BMI, or other adipokines. Participants were also classified according to Tanner stages as pre-/early pubertal (stage 1 and 2), pubertal (stage 3 and 4) and post-pubertal (stage 5) and they did not find any evidence for differences depending on pubertal status. However, the pubertal stages of these adolescents were not analyzed separately for males and females, which we believe is not accurate. Overall, circulating irisin levels at baseline increased by 12% after the one year exercise intervention for obesity. In the same study, no correlation was found between BMI standard deviation score and irisin changes.

Jang et al (21) evaluated the relationships between circulating irisin and metabolic profiles and anthropometric indices in adolescents between 12-15 years of age in two groups, one with healthy body weight and one with obesity. They found that circulating irisin was positively correlated with adiposity indices, including percent BFR, fat mass, and the ratio of fat mass to FFM. Again, girls had higher irisin levels than boys after adjusting for confounders in the normal-weight adolescents but not in the obese adolescents. In the same research adolescents were further classified as prepubertal (stage 1 and 2), pubertal (stage 3 and 4) and postpubertal (stage 5 and 6) in both normal weight and obese groups but again not differentiating for girls and boys and serum irisin levels did not differ significantly between the groups. They also analyzed the levels of irisin in two groups as pre-menarche or post-menarche in girls and did not find any difference. They found that elevated circulating irisin in adolescents was associated with obesity, whereas irisin increased in adolescents with healthy body weight after exercise but not in the obese group.

Lastly, Reinehr et al (23) investigated irisin and its relation to pubertal status in children and adolescents. Pubertal developmental status was categorized into two groups based on breast and genital stages determined according to Marshall and Tanner (prepubertal, boys with genital stage 1 and girls with breast stage 1; and pubertal, boys with genital stage ≥2 and girls with breast stage ≥2). The irisin concentrations differed significantly between the prepubertal and pubertal children. Analyzing only obese children demonstrated the same findings; the irisin concentrations differed significantly between the obese prepubertal and obese pubertal children.

While evaluating the changes of circulating irisin levels during puberty, it should not be overlooked that irisin is an adipomyokine (10) and its levels may depend on the ratio of body fat and muscle mass (9) which varies with gender and pubertal stage during adolescence. The levels of irisin in girls were already documented to be higher than boys (21,29) and we believe that comparing the irisin levels in the pre-early puberty versus mid-puberty versus post-puberty can only be done accurately by analyzing the data separately for females and males, which is not the case for the above studies (21,22). Relevantly, the study by Reinehr et al (23) has shown that the levels of irisin significantly increase with the onset of puberty in both sexes and the study by Jang et al (21) reported that irisin levels do not change in girls before and after menarche. Thus, we investigated the irisin levels in male adolescents after the onset of puberty, which we believe was a neglected area of irisin-puberty research.

We investigated circulating irisin levels only in boys, keeping the age constant (within 1.5 years) to avoid the confounding effect of age differences and to test the hypothesis that the irisin concentration may vary in the course of sexual maturation. The age 14 years (>13^6/12^ years) was chosen to exclude pre-pubertal boys and to have adolescent boys at different stages of pubertal development since normal puberty in boys would start before 13^6/12^ years of age. We also only included healthy boys with normal body weight that had not exercised the day before in order to ensure the only independent variable in grouping the participants of the study was the Tanner stage.

This study evaluated BMI, body fat by two different methods, namely BIA and triceps skinfold thickness and FFM of male adolescents together with irisin concentrations. The significant correlation found between Triceps-BFR and BIA-BFR validated the measurements by different methods. BMI and body fat mass percentages did not differ significantly between pubertal stages, whereas the FFM increased significantly with progressing stages. These results are in concordance with previous studies reporting that the lean body mass changes from approximately 80% of body weight in early puberty to 90% at maturity, which primarily reflects increased muscle mass in male adolescents while the percentage of body fat during puberty decreases from stage 1 to stage 2 and remains unchanged in stages 3, 4 and 5 in males (30,31). Also, in male adolescents, gain in muscle mass reaches its maximum velocity in accordance with the peak height velocity which occurs at Tanner Stage 4 in boys (32) and FFM increase in our study group was found to be maximum from stage 3 to 4. Thus, the size of our study population was large enough to document any physiological changes during pubertal development, if present.

However, we did not find any significant variation in irisin concentrations between pubertal stages. Correlation between irisin levels and BMI, body fat and FFM was investigated for the whole study population and further, separately in each pubertal stage. We did not find any correlations between any of these anthropometric parameters and irisin concentration.

### Study Limitations

Serum irisin was measured by a commercial ELISA method, and more reliable levels can be measured by immunoblotting (33) and mass spectroscopy (34), but still, ELISA method allows us to compare between the stages. Mass spectrometry method (34) is sophisticated and not available in every laboratory. It has been demonstrated by the immunoblotting method that the irisin can be measured by binding with the FDNC5 antibody (33). These methods have been preferred in early studies of irisin. In our study, we preferred the ELISA method, which is more widely used.

## Conclusion

Although recent findings indicate that irisin levels might differ between prepubertal and pubertal boys, the results of this study suggest that levels do not differ with progressing pubertal maturation in male adolescents.

## Figures and Tables

**Table 1:**
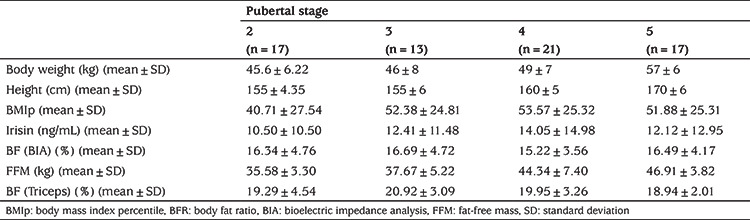
Body mass index percentile, irisin, body fat ratio (%) and fat-free mass values according to pubertal stages

**Table 2:**
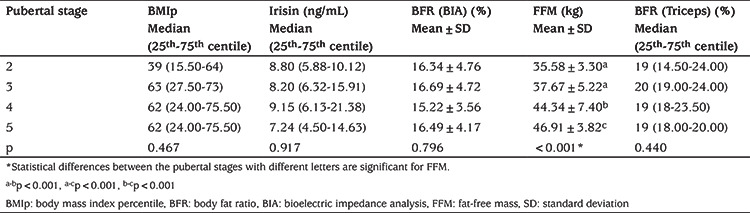
Statistical analysis of variables according to pubertal stage

**Table 3:**
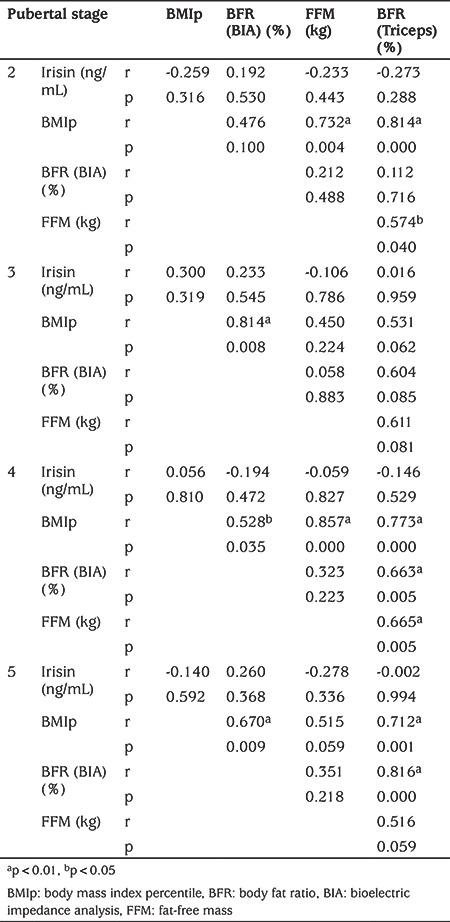
Correlation analysis of body mass index percentile, body fat ratio (BFR) (bioelectric impedance analysis) (%), fat-free mass (kg) and BFR (Triceps) (%) variables according to pubertal stages

**Table 4:**
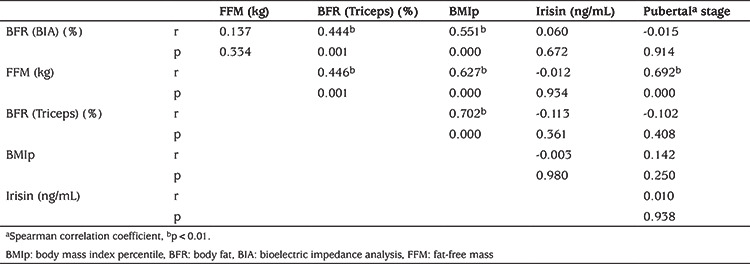
The inter-correlations between the parameters studied
